# Case Report: Clinical effect of combining auricular cartilage, full-thickness auricular skin graft, and local flap from the right nasal ala in complex nasal defect reconstruction

**DOI:** 10.3389/fsurg.2024.1440418

**Published:** 2024-09-13

**Authors:** Jinfu Zuo, Rong Wang, Xiaoting Fan, Haixia Zhang, Zhaohui Zhai, Huachang Sun

**Affiliations:** ^1^ Department of Plastic and Reconstructive Surgery, Zibo Central Hospital, Zibo, China; ^2^ Institute of Plastic Surgery, Shandong Second Medical University, Weifang, China

**Keywords:** nasal deformity, flap, prefabricated skin, ear cartilage, nasal defect

## Abstract

The aim of this report was to evaluate the efficacy of nasal reconstruction using auricular cartilage combined with auricular full-thickness skin graft and a local flap from the right nasal ala for correcting complex nasal defects, and its impact on the patient's quality of life and psychological well-being. We present the case of a 50-year-old female with a severe nasal deformity due to a car accident, characterized by a missing right alar flap and an exposed right nostril. The patient underwent nasal reconstructive surgery using auricular cartilage combined with an auricular full-thickness skin graft and a local flap from the right nasal ala. Detailed records of the patient's postoperative recovery, surgical outcomes, and satisfaction were made at follow-up. The survival rate of the grafted skin and flaps on the nasal ala, septum, and dorsum was high. The reconstructed right nasal ala closely resembled the left, and the patient was very satisfied. In patients with complex nasal deformities, nasal reconstruction using ear cartilage combined with auricular full-thickness skin grafts and local flaps can achieve satisfactory aesthetic and functional outcomes. The high level of postoperative satisfaction suggests that this technique significantly improves patients’ quality of life and psychological well-being.

## Introduction

The nose, centrally located on the face, is a vital structure with a complex three-dimensional anatomy crucial for maintaining both facial aesthetics and respiratory function (1). Nasal deformities can be broadly classified into congenital and acquired categories. Congenital anomalies, such as cleft lip and palate, can result in nasal deformities, while acquired conditions may arise from trauma injuries or surgical interventions. Regardless of etiology, nasal deformities can profoundly affect a patient's quality of life and psychological well-being. In cases of external nasal deformities, patients often have high expectations for aesthetic and functional reconstruction. Treatment goals extend beyond restoring nasal function to include the meticulous reshaping to achieve the nose's pre-injury form, ensuring symmetry, and meeting aesthetic standards (2).

Currently, treatment methods vary depending on the underlying cause of nasal deformities. In this article, we present a clinical case of a female patient who suffered a nasal deformity due to a car accident. Given the patient's specific nasal deformity and reconstructive requirements, we employed a combined approach using auricular cartilage grafts along with an auricular full-thickness skin graft and a local flap from the right nasal ala to repair the alar defect.

### Patient and methods

**Figure 1:**
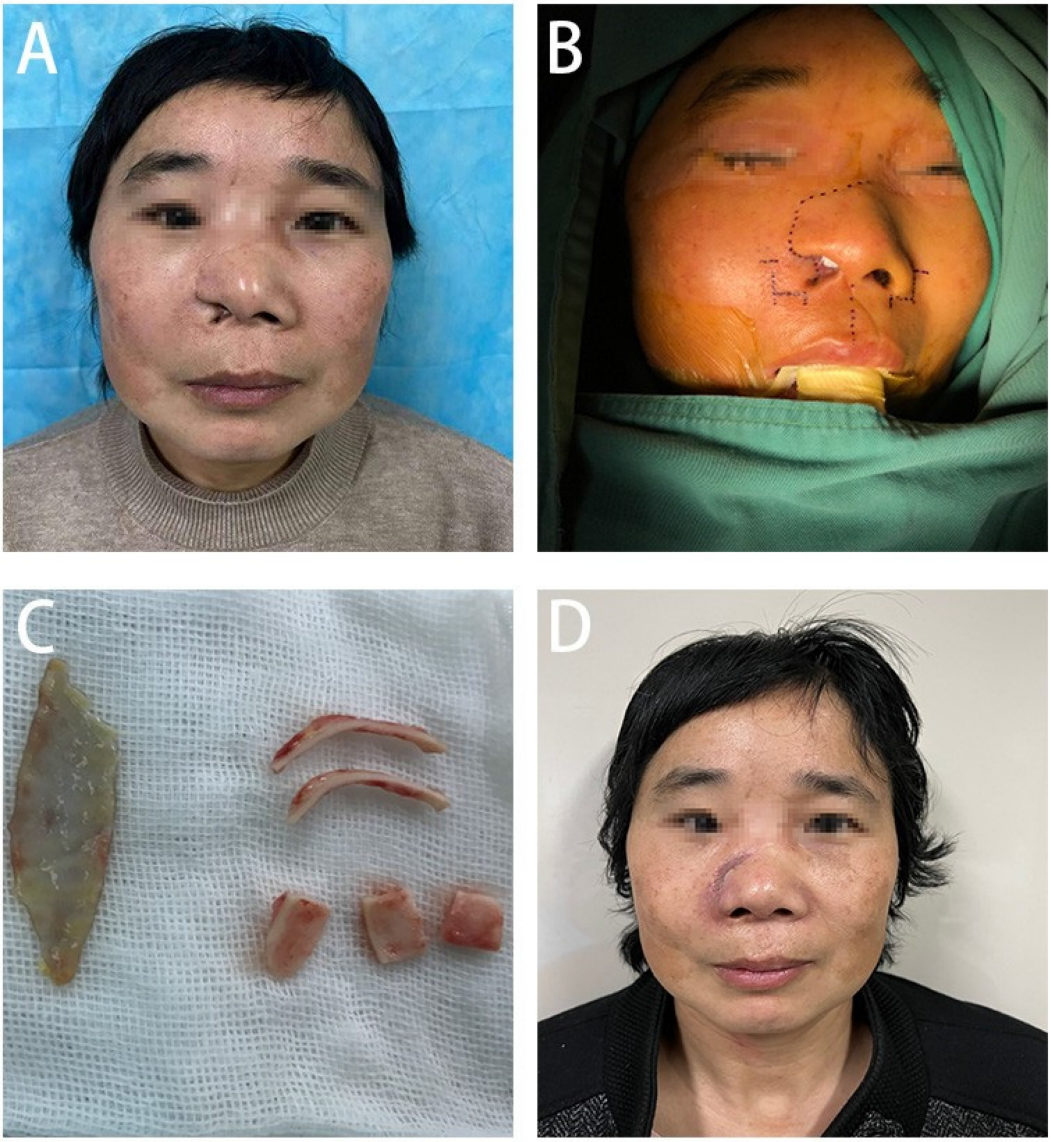
**(A)** Presents the pre-surgical condition of the patient's nasal structural deformity, **(B)** shows in detail the precise incision design for nasal reconstruction, **(C)** demonstrates the process of extracting and preparing the cartilage scaffold from the patient's ear, and **(D)** depicts the patient's nasal recovery one month after undergoing the surgical treatment.

Two years ago, a 50-year-old female patient sustained multiple facial lacerations due to a vehicular accident, with the most severe injury localized to the right side of her face. Despite receiving emergency surgical intervention, the healing process resulted in significant scarring, leading to the loss of the right nasal ala, external nostril retraction, and a marked local deformity (Figure 1A). These outcomes notably impaired her physical appearance and psychosocial well-being.

**Figure 2:**
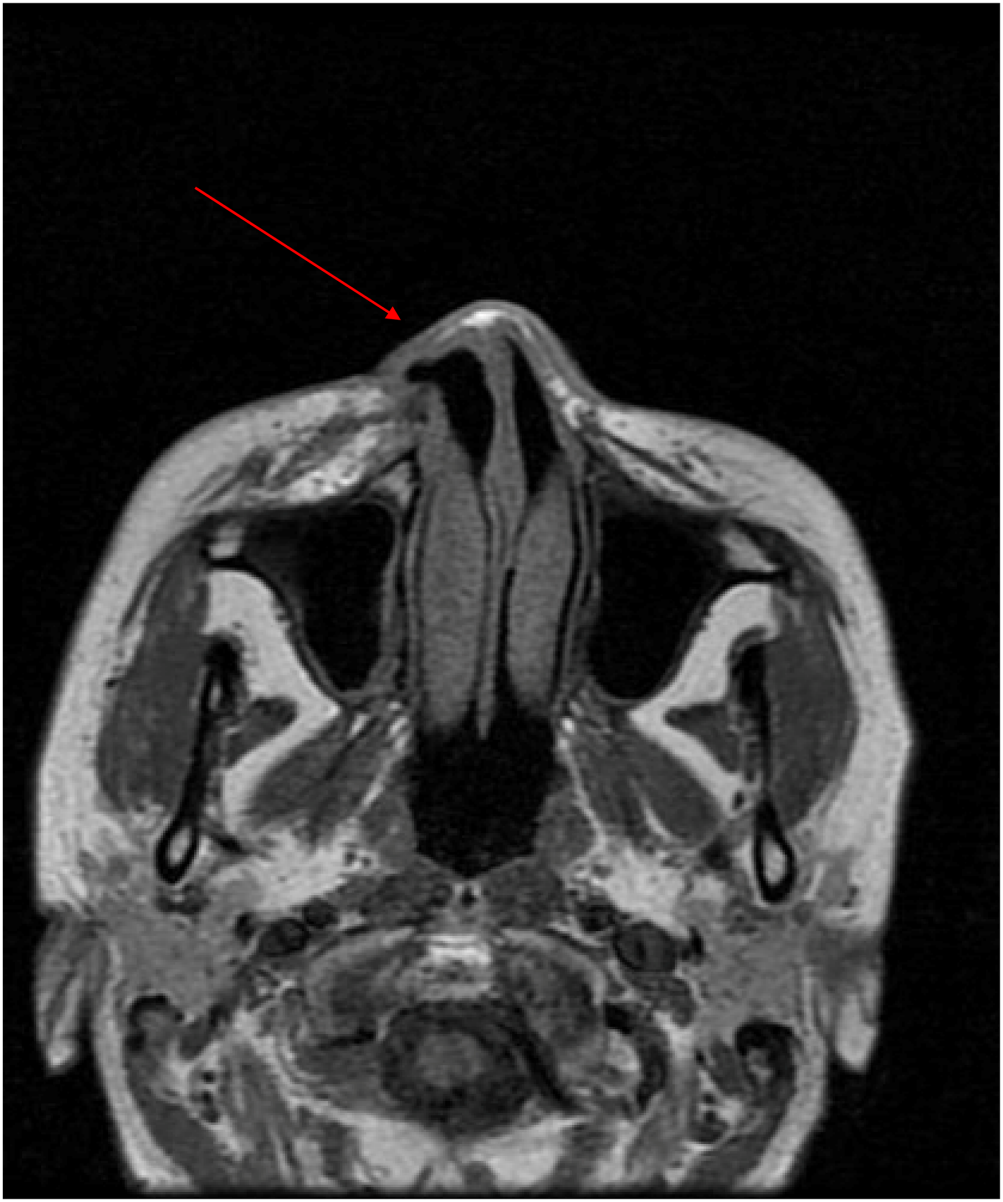
Right nasal soft tissue discontinuity and slight thickening of the dorsal nasal and maxillofacial soft tissues.

Preoperative evaluation revealed post-traumatic scars traversing diagonally across the right forehead, nose, and upper lip. The defect in the right nasal ala, involving both skin and cartilage, measured approximately 2 × 1 cm and was accompanied by nostril retraction, extensive scarring, and tissue tension. Facial magnetic resonance imaging (MRI) revealed a soft tissue discontinuity in the right nasal ala and mild soft tissue hypertrophy within the nasal dorsum and maxillofacial area (Figure 2). During the preoperative preparation phase, detailed anatomical marking and precise surgical labeling were meticulously performed to ensure clarity and accuracy in the surgical plan for the reconstruction (Figure 1B).

Under general anesthesia, the patient underwent a right nasal reconstruction procedure that included a local flap transfer from the right nasal ala, a full-thickness postauricular skin graft, and auricular cartilage grafting. An incision was made along the pre-existing wound, and the dorsal nasal tissue was widely dissected to create a cutaneous flap. The flap was extended by retracting it towards the cephalic direction to the base of the nose, and hemostasis was achieved after measuring the appropriate length. Tumescent solution was injected inside and outside the right conchal bowl, and an incision was made along the medial edge to separate the auricular cartilage. Approximately 3 × 2 cm of auricular cartilage was harvested for later use. After hemostasis, the incision was sutured and secured with petrolatum gauze. Tumescent solution was injected behind the left ear, and the skin was incised along the postauricular sulcus. A full-thickness skin graft measuring about 5 × 3 cm was harvested for later use. The incision was closed after hemostasis.

The nasal cartilage was shaped by cutting two bowed strips (about 2.5 cm × 1 cm), one shield-shaped piece (about 2 cm × 1.2 cm), and two dome-shaped pieces (about 1.5 cm × 1.5 cm), which were placed at the nasal alae, columella, and tip of the nose, respectively, and trimmed to the appropriate size based on preoperative measurements and symmetry to achieve bilateral symmetry (Figure 1C). The position of each piece was adjusted and properly secured. The dorsal tissue flap was advanced to reconstruct the outer layer of the nose, and an internal graft was placed and fixed on the inner side of the nasal cavity. A full-thickness skin graft was transplanted and fixed at the dorsal nasal defect. After confirming that the shape was satisfactory, nasal support was provided, and the outer layer was properly bandaged.

During the one-month postoperative evaluation, significant improvement in the nasal profile was noted, with the patient expressing considerable satisfaction with the surgical outcome (Figure 1D).

## Discussion

The origins of nasal reconstruction date back over 3,000 years to ancient India and are largely attributed to the pioneering work of Sushruta. Sushruta's methodologies were remarkably innovative for their time, employing cheek skin flaps for nasal reconstruction, setting the foundation for future techniques. As centuries passed, the discipline of nasal reconstruction gradually evolved, with the development of various techniques and approaches. A significant milestone in this journey was the introduction of the “Italian method” by Gaspare Tagliacozzi, which utilized a pedicled flap from the upper arm for nasal reconstruction. In contemporary nasal reconstruction, the use of synthetic implants for nasal augmentation, a technique widely attributed to Nishihata, marks a modern advancement in the field (3).

Nasal deformities pose a frequent and significant challenge in plastic surgery. Although nasal defects may not cover a large area, their central position on the face and the nose's unique three-dimensional structure and function make repair inherently complex. Achieving satisfactory aesthetic outcomes after nasal reconstruction is challenging due to the intricate anatomy involved. The goals of nasal defect repair include restoring function, enhancing aesthetics, and prioritizing the nose's appearance to achieve an ideal reconstructive result. In reconstructive surgery, repairing nasal deformities typically involves addressing the nasal lining, structural framework, and external covering (3). The specific surgical approach should be tailored to the circumstances of each patient.

In this case, the primary issue was a right alar deformity accompanied by skin and cartilage loss, exposure of the nostril, and surrounding tissue scarring and adhesions. Considering the patient's condition, we employed a surgical approach involving alar reconstruction using auricular cartilage combined with a full-thickness postauricular skin graft and a local flap from the right nasal ala. The auricular cartilage was used to rebuild the structural support, the postauricular skin graft provided the necessary internal lining, and the local nasal flap was utilized for external coverage to restore the contour and symmetry of the nasal ala.

There are several options for flap selection in reconstructive surgery, including the auricular composite tissue flap. Its advantages include maintaining relatively intact functionality and having a color, texture, and thickness similar to nasal skin. Additionally, auricular cartilage can serve as a framework to address alar cartilage defects. This makes it suitable for full-thickness alar defects (4). However, vascular anastomosis can be relatively complex, with a lower survival rate. Adequate blood supply from surrounding tissues is crucial. Additionally, excessive tissue harvesting can lead to ischemic necrosis of the flap.

Local flaps, such as rhomboid flaps, are suitable for small isolated alar defects with a diameter of less than 2 centimeters. Their advantages include color matching with the recipient site, similar histological characteristics, and being less technically demanding. However, they can cause issues such as donor site morbidity, multiple incisions, and residual scarring (1, 5). The forehead flap is suitable for a wide range of nasal defect repairs and can even be used for total nasal reconstruction. Forehead skin is dense and resilient, with a rich blood supply, closely matching the color, texture, and thickness of nasal skin, thus providing a stable postoperative appearance. However, scarring is common at the donor site, making it unsuitable for patients with existing forehead scars. Patients with a low hairline may experience limited flap length in the donor area, and if the selected flap extends into the hair, depilation may be required. Additionally, the forehead flap has potential complications such as bleeding, pain, infection, and necrosis (6, 7). The folded nasolabial flap is also an option, with advantages such as similar color, shorter operation time, avoidance of secondary surgery, and lining repair. However, it also presents challenges such as facial scarring, flap necrosis, and the potential need for additional shape correction (8). Postauricular flaps can be used for repairing large alar defects and reconstructing nasal columella defects. The primary blood supply for postauricular flaps comes from the superficial temporal artery and the postauricular artery. Their advantages include a relatively hidden donor site behind the ear, which offers favorable aesthetic outcomes. However, there is a risk of insufficient vascularization, potentially leading to necrosis (9).

Based on the patient's specific condition, we selected a local flap from the right nasal ala for alar defect repair. This approach allowed us to reconstruct the alar defect while preserving its original structure, function, appearance, texture, and color. For the internal lining of the nasal defect, a full-thickness postauricular skin graft was employed. Ensuring the vascularity of the flap during surgery is essential, and careful dissection and mobilization of the surrounding nasal tissue were performed to minimize tension and ensure a secure attachment.

Additionally, postoperative contouring is necessary to prevent complications such as contracture. The choice of lining repair is a crucial aspect of nasal defect reconstruction. Inadequate lining repair can lead to nasal cavity contracture, affecting nasal ventilation function. An ideal nasal lining should possess the following characteristics: 1. Adequate vascularization: Sufficient blood supply is fundamental for flap viability. 2. Soft and thin texture: The lining should be soft and thin, closely resembling the normal physiological structure of the nasal cavity, to prevent obstruction and maintain airway function. 3. Reduced tension: Minimizing tension is crucial to avoid postoperative contracture (10–12).

Various options for nasal lining repair include local tissue flap rotation, mucosal flaps, and free grafts (13). When conditions allow, local tissue flap rotation is a favorable choice because it causes minimal trauma, is simple to perform, and closely matches the physiological properties of the nasal lining. However, this technique is best suited for minimal lining defects with well-vascularized surrounding tissues. Additionally, it may lead to hypertrophic repair areas.

Mucosal flaps closely resemble the normal physiological structure of the nasal lining, but they involve complex surgical procedures and pose significant challenges in tissue procurement. They are not suitable for repairing large defects (14, 15). In this case, we opted for postauricular free skin graft transplantation for lining repair. This involves harvesting a free skin graft from the postauricular region, transplanting it into the inner nasal cavity, and securing it to restore the nasal lining. We chose a moderately thick graft because it offers appropriate thickness and pliability for the reconstructed lining and has comparatively high survival rates. Postoperative shrinkage and color changes are less pronounced, and there are various donor site options.

Support structures are crucial for maintaining the external shape and airflow function of the nose. Inadequate support can lead to deformities such as open roof deformities and inverted V deformities (16). Currently, options for nasal support structures typically include autologous materials such as auricular cartilage and costal cartilage, as well as synthetic materials (17). Synthetic materials are derived from various sources and do not require harvesting from the patient's own body, thus avoiding donor site morbidity and the limitations of donor tissue availability. The surgical procedure using synthetic materials is relatively straightforward; however, they carry a higher risk of postoperative infection. As a result, synthetic materials are generally not the primary choice unless there is a significant shortage of autologous tissue.

Autologous rib cartilage is a relatively abundant source that provides excellent support. However, it lacks elasticity, has limited shaping ability, and poses a risk of calcification, particularly in elderly patients (18). In contrast, ear cartilage is highly valued for its excellent pliability and malleability, making it ideal for reconstructing both the external appearance and internal structure of the nose. Therefore, ear cartilage was chosen for nasal support in this specific case.

In summary, to repair the nasal deformity, we utilized a local flap from the right nasal ala for external coverage, a full-thickness postauricular skin graft for internal lining, and auricular cartilage for structural support. Postoperatively, the patient exhibited excellent viability of the nasal flap, effective incorporation of the skin graft within the nasal cavity, adequate mucosal lining function, and satisfactory contouring of the support structure. At the 1-month postoperative mark (Figure 1D), there was significant improvement in the patient's nasal aesthetics. The patient expressed a high level of satisfaction with the outcome, accompanied by a notable improvement in quality of life.

## Outlook

Recent advancements in tissue engineering have led to the adoption of 3D printing technology across various domains, particularly in organ reconstruction. A particularly innovative application has been the development of nasal support structures using mesenchymal stem cells via 3D printing. This technique not only mitigates the challenges associated with tissue source scarcity but also minimizes the risks of rejection and infection. Researchers such as Fulco et al. have pioneered the application of tissue engineering in nasal reconstruction, charting new paths for nasal repair methodologies (19, 20).

As the field of reconstructive surgery advances, an expanding array of surgical techniques for nasal defect and deformity repair is emerging, each with its own set of advantages and limitations. The selection of the most suitable surgical approach must be tailored to the individual requirements of each patient. It is anticipated that ongoing technological advancements will further enhance and refine nasal repair procedures.

## Data Availability

The original contributions presented in the study are included in the article/Supplementary Material, further inquiries can be directed to the corresponding author.
